# 2-Chloro-*N*-(2,6-dichloro­phen­yl)benzamide

**DOI:** 10.1107/S1600536808021223

**Published:** 2008-07-16

**Authors:** B. Thimme Gowda, Miroslav Tokarčík, Jozef Kožíšek, B. P. Sowmya, Hartmut Fuess

**Affiliations:** a Department of Chemistry, Mangalore University, Mangalagangotri 574 199, Mangalore, India; b Faculty of Chemical and Food Technology, Slovak Technical University, Radlinského 9, SK-812 37 Bratislava, Slovak Republic; c Institute of Materials Science, Darmstadt University of Technology, Petersenstrasse 23, D-64287 Darmstadt, Germany

## Abstract

In the structure of the title compound (N26DCP2CBA), C_13_H_8_Cl_3_NO, the conformations of N—H and C=O bonds in the amide group are *trans* to each other, similar to that observed in *N*-(2,6-dichloro­phen­yl)benzamide, 2-chloro-*N*-phenyl­benzamide, 2-chloro-*N*-(2-chloro­phen­yl)benzamide and 2-chloro-*N*-(2,3-dichloro­phen­yl)benzamide with similar bond parameters. Furthermore, the position of the amide O atom is *syn* to the *ortho*-chloro group in the benzoyl ring. The amide group makes a dihedral angle of 59.8 (1)° with the benzoyl ring, while the benzoyl and aniline rings make a dihedral angle of 8.1 (2)°. The mol­ecules are linked by N—H⋯O hydrogen bonds into infinite chains running along the *b* axis.

## Related literature

For related literature, see Gowda *et al.* (2003 ▶, 2007 ▶, 2008*a* ▶,*b* ▶).
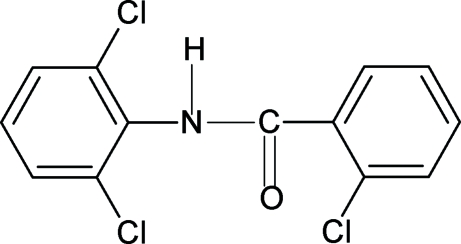


## Experimental

### 

#### Crystal data

C_13_H_8_Cl_3_NO
*M* *_r_* = 300.55
Orthorhombic, 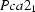

*a* = 21.3949 (4) Å
*b* = 4.8159 (1) Å
*c* = 12.5036 (3) Å
*V* = 1288.32 (5) Å^3^
*Z* = 4
Mo *K*α radiation
μ = 0.70 mm^−1^
*T* = 295 (2) K
0.42 × 0.16 × 0.08 mm

#### Data collection

Oxford Diffraction Xcalibur System diffractometer
Absorption correction: analytical [*CrysAlis RED*; Oxford Diffraction, 2007 ▶ (based on Clark & Reid, 1995 ▶)] *T*_min_ = 0.802, *T*_max_ = 0.951
26609 measured reflections
1306 independent reflections
1216 reflections with *I* > 2σ(*I*)
*R*_int_ = 0.030

#### Refinement

*R*[*F* ^2^ > 2σ(*F* ^2^)] = 0.027
*wR*(*F* ^2^) = 0.073
*S* = 1.08
1306 reflections
163 parameters
1 restraint
H-atom parameters constrained
Δρ_max_ = 0.20 e Å^−3^
Δρ_min_ = −0.21 e Å^−3^
Absolute structure: Flack (1983 ▶), 1167 Friedel pairs
Flack parameter: 0.14 (8)

### 

Data collection: *CrysAlis CCD* (Oxford Diffraction, 2007 ▶); cell refinement: *CrysAlis RED* (Oxford Diffraction, 2007 ▶); data reduction: *CrysAlis RED*; program(s) used to solve structure: *SHELXS97* (Sheldrick, 2008 ▶); program(s) used to refine structure: *SHELXL97* (Sheldrick, 2008 ▶); molecular graphics: *ORTEP-3* (Farrugia, 1997 ▶) and *DIAMOND* (Brandenburg, 2002 ▶); software used to prepare material for publication: *SHELXL97*, *PLATON* (Spek, 2003 ▶) and *WinGX* (Farrugia, 1999 ▶).

## Supplementary Material

Crystal structure: contains datablocks I, global. DOI: 10.1107/S1600536808021223/bx2156sup1.cif

Structure factors: contains datablocks I. DOI: 10.1107/S1600536808021223/bx2156Isup2.hkl

Additional supplementary materials:  crystallographic information; 3D view; checkCIF report

## Figures and Tables

**Table 1:** Hydrogen-bond geometry (Å, °)

*D*—H⋯*A*	*D*—H	H⋯*A*	*D*⋯*A*	*D*—H⋯*A*
N1—H1*N*⋯O1^i^	0.86	2.02	2.840 (3)	158

Symmetry code: (i) 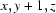.

**Fig. 1.**
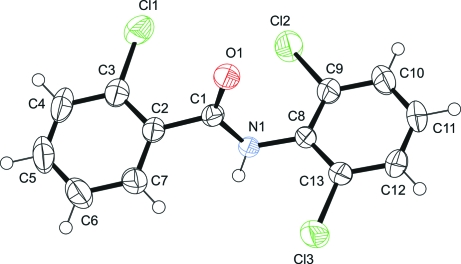
Molecular structure of the title compound showing the atom labelling scheme. The displacement ellipsoids are drawn at the 50% probability level. The H atoms are represented as small spheres of arbitrary radii.

**Fig. 2.**
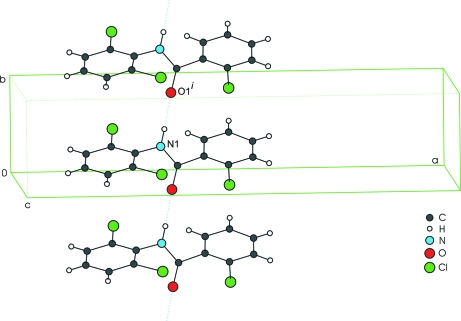
Part of the crystal structure of the title compound with infinite molecular chains running along the b axis of the crystal. The chains are generated by N—H···O(i) hydrogen bonds. Symmetry operation (i):x,y + 1,z.

## supplementary crystallographic information

### Comment

In the present work, the structure of 2-chloro-*N*-(2,6-dichlorophenyl)- benzamide (N26DCP2CBA) has been determined to explore the effect of substituents on the structures of benzanilides (Gowda *et al.*, 2003; 2007; 2008*a,b*). The N—H and C═O bonds in the amide group of N26DCP2CBA are *trans* to each other (Fig.1), similar to that observed in *N*-(2,6-dichlorophenyl)benzamide (Gowda *et al.*, 2008*b*), 2-chloro-*N*-(phenyl)-benzamide (NP2CBA)(Gowda *et al.*, 2003), 2-chloro-*N*-(2-chlorophenyl)-benzamide (Gowda *et al.*, 2007), 2-chloro-*N*-(2,3-dichlorophenyl)benzamide (Gowda *et al.*, 2008*a*), 2-chloro-*N*-(3,5-dichlorophenyl)-benzamide (N35DCP2CBA) (Gowda *et al.*, 2008*a*) and other benzanilides. Further, the conformation of the amide oxygen in N26DCP2CBA is *syn* to the *ortho*-chloro group in the benzoyl ring, similar to that observed in NP2CBA. The amide group –NHCO– makes dihedral angle of 59.8 (1)° with the benzoyl ring, while the benzoyl and aniline rings make dihedral angle of 8.1 (2)°), compared to the corresponding dihedral angles of 63.1 (12)° and 32.1 (2)°) observed in N35DCP2CBA.

Part of the crystal structure of N26DCP2CBA with infinite molecular chains running along the *b* axis of the crystal is shown in Fig. 2. The chains are generated by N—H···O(i) hydrogen bonds (Table 1). Symmetry operation (i):*x*,*y* + 1,*z*.

### Experimental

The title compound was prepared according to the method of Gowda *et al.*, (2003). The purity of the compound was checked by determining its melting point. It was characterized by recording its infrared and NMR spectra. Single crystals of the title compound used in X-ray diffraction studies were obtained from a slow evaporation of an ethanolic solution at room temperature.

### Refinement

All H atoms were placed in calculated positions and subsequently treated as riding with C–H distance of 0.93Å and N–H distance of 0.86 Å. The *U*_iso_(H) values were set at 1.2 *U*_eq_(C,*N*).

### Crystal data

**Table:**

C_13_H_8_Cl_3_NO	*F*_000_ = 608
*M**_r_* = 300.55	*D*_x_ = 1.55 Mg m^−^^3^
Orthorhombic, *P**c**a*2_1_	Mo *K*α radiation λ = 0.71073 Å
Hall symbol: P 2c -2ac	Cell parameters from 13276 reflections
*a* = 21.3949 (4) Å	θ = 3.3–29.5º
*b* = 4.81590 (10) Å	µ = 0.70 mm^−^^1^
*c* = 12.5036 (3) Å	*T* = 295 (2) K
*V* = 1288.32 (5) Å^3^	Rod, colourless
*Z* = 4	0.42 × 0.16 × 0.08 mm

### Data collection

**Table:**

Oxford Diffraction Xcalibur System diffractometer	1306 independent reflections
Monochromator: graphite	1216 reflections with *I* > 2σ(*I*)
Detector resolution: 10.434 pixels mm^-1^	*R*_int_ = 0.030
*T* = 295(2) K	θ_max_ = 26.0º
ω scans with κ offsets	θ_min_ = 5.3º
Absorption correction: analytical(CrysAlis RED; Oxford Diffraction, 2007)	*h* = −26→26
*T*_min_ = 0.802, *T*_max_ = 0.951	*k* = −5→5
26609 measured reflections	*l* = −15→15

### Refinement

**Table:**

Refinement on *F*^2^	Hydrogen site location: inferred from neighbouring sites
Least-squares matrix: full	H-atom parameters constrained
*R*[*F*^2^ > 2σ(*F*^2^)] = 0.027	*w* = 1/[σ^2^(*F*_o_^2^) + (0.044*P*)^2^ + 0.2234*P*] where *P* = (*F*_o_^2^ + 2*F*_c_^2^)/3
*wR*(*F*^2^) = 0.073	(Δ/σ)_max_ < 0.001
*S* = 1.08	Δρ_max_ = 0.21 e Å^−^^3^
1306 reflections	Δρ_min_ = −0.21 e Å^−^^3^
163 parameters	Extinction correction: none
1 restraint	Absolute structure: Flack (1983), 1167 Friedel pairs
Primary atom site location: structure-invariant direct methods	Flack parameter: 0.14 (8)
Secondary atom site location: difference Fourier map	

### Special details

**Table:**

Experimental. CrysAlis RED, Oxford Diffraction (2007). Analytical absorption correction using a multifaceted crystal model based on expressions derived by Clark & Reid (1995).
Geometry. All e.s.d.'s (except the e.s.d. in the dihedral angle between two l.s. planes) are estimated using the full covariance matrix. The cell e.s.d.'s are taken into account individually in the estimation of e.s.d.'s in distances, angles and torsion angles; correlations between e.s.d.'s in cell parameters are only used when they are defined by crystal symmetry. An approximate (isotropic) treatment of cell e.s.d.'s is used for estimating e.s.d.'s involving l.s. planes.
Refinement. Refinement of *F*^2^ against ALL reflections. The weighted *R*-factor *wR* and goodness of fit *S* are based on *F*^2^, conventional *R*-factors *R* are based on *F*, with *F* set to zero for negative *F*^2^. The threshold expression of *F*^2^ > σ(*F*^2^) is used only for calculating *R*-factors(gt) *etc*. and is not relevant to the choice of reflections for refinement. *R*-factors based on *F*^2^ are statistically about twice as large as those based on *F*, and *R*- factors based on ALL data will be even larger.

### Fractional atomic coordinates and isotropic or equivalent isotropic displacement parameters (Å^2^)

**Table:**

	*x*	*y*	*z*	*U*_iso_*/*U*_eq_	
C1	0.36930 (11)	0.1547 (5)	0.4253 (2)	0.0334 (5)	
C2	0.42847 (11)	0.2602 (5)	0.4746 (2)	0.0357 (6)	
C3	0.48616 (13)	0.1593 (6)	0.4430 (3)	0.0429 (7)	
C4	0.54063 (13)	0.2457 (7)	0.4927 (3)	0.0579 (9)	
H4	0.5791	0.1756	0.4709	0.069*	
C5	0.53744 (18)	0.4341 (8)	0.5737 (4)	0.0687 (12)	
H5	0.5739	0.4915	0.6075	0.082*	
C6	0.48090 (19)	0.5403 (8)	0.6060 (3)	0.0634 (11)	
H6	0.4792	0.6695	0.6612	0.076*	
C7	0.42636 (16)	0.4543 (6)	0.5560 (3)	0.0481 (8)	
H7	0.3881	0.5275	0.5775	0.058*	
C8	0.27485 (10)	0.2660 (5)	0.3315 (2)	0.0334 (5)	
C9	0.27249 (14)	0.1003 (6)	0.2411 (3)	0.0470 (7)	
C10	0.21674 (15)	0.0145 (9)	0.1969 (3)	0.0609 (10)	
H10	0.2164	−0.0982	0.1365	0.073*	
C11	0.16185 (15)	0.0976 (7)	0.2434 (3)	0.0592 (9)	
H11	0.124	0.0388	0.2144	0.071*	
C12	0.16174 (12)	0.2649 (7)	0.3313 (3)	0.0491 (7)	
H12	0.1242	0.322	0.3618	0.059*	
C13	0.21824 (12)	0.3488 (6)	0.3745 (3)	0.0389 (6)	
N1	0.33226 (9)	0.3444 (4)	0.37870 (19)	0.0337 (5)	
H1N	0.3436	0.5157	0.3775	0.04*	
O1	0.35555 (10)	−0.0906 (4)	0.4302 (2)	0.0510 (6)	
Cl1	0.49200 (4)	−0.07634 (17)	0.33767 (8)	0.0588 (2)	
Cl2	0.34152 (4)	0.0008 (2)	0.17928 (9)	0.0734 (3)	
Cl3	0.21723 (4)	0.5602 (2)	0.48512 (8)	0.0670 (3)	

### Atomic displacement parameters (Å^2^)

**Table:**

	*U*^11^	*U*^22^	*U*^33^	*U*^12^	*U*^13^	*U*^23^
C1	0.0284 (12)	0.0289 (12)	0.0429 (14)	0.0002 (10)	0.0006 (11)	0.0002 (11)
C2	0.0316 (12)	0.0281 (12)	0.0473 (15)	−0.0025 (10)	−0.0049 (12)	0.0079 (11)
C3	0.0363 (14)	0.0375 (15)	0.0547 (18)	−0.0027 (11)	−0.0038 (13)	0.0107 (13)
C4	0.0299 (14)	0.0577 (19)	0.086 (2)	−0.0036 (13)	−0.0126 (16)	0.018 (2)
C5	0.051 (2)	0.060 (2)	0.095 (3)	−0.0124 (17)	−0.039 (2)	0.010 (2)
C6	0.073 (2)	0.051 (2)	0.067 (3)	−0.0040 (18)	−0.032 (2)	−0.0082 (17)
C7	0.0501 (18)	0.0384 (15)	0.0558 (19)	0.0023 (13)	−0.0142 (15)	−0.0031 (14)
C8	0.0289 (11)	0.0280 (11)	0.0435 (14)	−0.0012 (9)	−0.0038 (11)	−0.0033 (12)
C9	0.0329 (14)	0.0548 (17)	0.0532 (18)	0.0029 (12)	−0.0013 (12)	−0.0146 (15)
C10	0.0459 (18)	0.073 (2)	0.064 (2)	−0.0028 (15)	−0.0125 (17)	−0.0282 (19)
C11	0.0336 (16)	0.067 (2)	0.077 (2)	−0.0063 (14)	−0.0159 (15)	−0.0159 (19)
C12	0.0267 (12)	0.0532 (17)	0.068 (2)	0.0012 (12)	−0.0004 (14)	−0.0072 (18)
C13	0.0347 (13)	0.0348 (14)	0.0471 (16)	−0.0002 (10)	−0.0009 (11)	−0.0076 (12)
N1	0.0284 (10)	0.0227 (9)	0.0498 (13)	−0.0009 (8)	−0.0055 (9)	−0.0042 (9)
O1	0.0432 (11)	0.0228 (9)	0.0870 (17)	−0.0051 (8)	−0.0100 (11)	0.0027 (10)
Cl1	0.0449 (4)	0.0632 (5)	0.0683 (5)	0.0093 (3)	0.0088 (4)	−0.0048 (4)
Cl2	0.0437 (4)	0.1065 (7)	0.0700 (6)	0.0068 (4)	0.0051 (4)	−0.0439 (5)
Cl3	0.0447 (4)	0.0801 (6)	0.0762 (6)	0.0001 (4)	0.0058 (4)	−0.0413 (5)

### Geometric parameters (Å, °)

**Table:**

C1—O1	1.219 (3)	C8—C13	1.384 (4)
C1—N1	1.343 (3)	C8—C9	1.385 (4)
C1—C2	1.497 (3)	C8—N1	1.414 (3)
C2—C7	1.383 (4)	C9—C10	1.378 (4)
C2—C3	1.384 (4)	C9—Cl2	1.734 (3)
C3—C4	1.385 (4)	C10—C11	1.370 (5)
C3—Cl1	1.743 (4)	C10—H10	0.93
C4—C5	1.361 (6)	C11—C12	1.363 (5)
C4—H4	0.93	C11—H11	0.93
C5—C6	1.374 (6)	C12—C13	1.384 (4)
C5—H5	0.93	C12—H12	0.93
C6—C7	1.387 (5)	C13—Cl3	1.717 (3)
C6—H6	0.93	N1—H1N	0.86
C7—H7	0.93		
			
O1—C1—N1	122.6 (2)	C13—C8—C9	116.8 (2)
O1—C1—C2	120.9 (2)	C13—C8—N1	121.4 (3)
N1—C1—C2	116.5 (2)	C9—C8—N1	121.7 (2)
C7—C2—C3	118.5 (3)	C10—C9—C8	122.1 (3)
C7—C2—C1	120.3 (3)	C10—C9—Cl2	118.4 (3)
C3—C2—C1	121.1 (3)	C8—C9—Cl2	119.5 (2)
C2—C3—C4	121.1 (3)	C11—C10—C9	119.0 (3)
C2—C3—Cl1	120.6 (2)	C11—C10—H10	120.5
C4—C3—Cl1	118.3 (3)	C9—C10—H10	120.5
C5—C4—C3	119.5 (3)	C12—C11—C10	121.1 (3)
C5—C4—H4	120.3	C12—C11—H11	119.5
C3—C4—H4	120.3	C10—C11—H11	119.5
C4—C5—C6	120.7 (3)	C11—C12—C13	119.1 (3)
C4—C5—H5	119.6	C11—C12—H12	120.5
C6—C5—H5	119.6	C13—C12—H12	120.5
C5—C6—C7	119.8 (3)	C8—C13—C12	121.9 (3)
C5—C6—H6	120.1	C8—C13—Cl3	119.6 (2)
C7—C6—H6	120.1	C12—C13—Cl3	118.5 (2)
C2—C7—C6	120.4 (3)	C1—N1—C8	120.8 (2)
C2—C7—H7	119.8	C1—N1—H1N	119.6
C6—C7—H7	119.8	C8—N1—H1N	119.6
			
O1—C1—C2—C7	−118.3 (3)	C13—C8—C9—Cl2	177.2 (2)
N1—C1—C2—C7	60.1 (3)	N1—C8—C9—Cl2	−3.5 (4)
O1—C1—C2—C3	59.2 (4)	C8—C9—C10—C11	0.6 (6)
N1—C1—C2—C3	−122.4 (3)	Cl2—C9—C10—C11	−178.3 (3)
C7—C2—C3—C4	1.2 (4)	C9—C10—C11—C12	0.7 (7)
C1—C2—C3—C4	−176.4 (3)	C10—C11—C12—C13	−0.7 (6)
C7—C2—C3—Cl1	−177.7 (2)	C9—C8—C13—C12	1.7 (5)
C1—C2—C3—Cl1	4.7 (4)	N1—C8—C13—C12	−177.6 (3)
C2—C3—C4—C5	−0.3 (5)	C9—C8—C13—Cl3	−178.7 (2)
Cl1—C3—C4—C5	178.6 (3)	N1—C8—C13—Cl3	2.0 (4)
C3—C4—C5—C6	−0.4 (6)	C11—C12—C13—C8	−0.5 (5)
C4—C5—C6—C7	0.3 (6)	C11—C12—C13—Cl3	179.9 (3)
C3—C2—C7—C6	−1.3 (4)	O1—C1—N1—C8	−0.5 (4)
C1—C2—C7—C6	176.2 (3)	C2—C1—N1—C8	−178.9 (3)
C5—C6—C7—C2	0.6 (5)	C13—C8—N1—C1	112.2 (3)
C13—C8—C9—C10	−1.7 (5)	C9—C8—N1—C1	−67.0 (4)
N1—C8—C9—C10	177.5 (3)		

### Hydrogen-bond geometry (Å, °)

**Table:**

*D*—H···*A*	*D*—H	H···*A*	*D*···*A*	*D*—H···*A*
N1—H1N···O1^i^	0.86	2.02	2.840 (3)	158

Symmetry codes: (i) *x*, *y*+1, *z*.
